# A Pathological Study of the Epidemiology of Atherosclerosis in Mexico City

**DOI:** 10.1155/2014/264205

**Published:** 2014-02-26

**Authors:** Joel Rodríguez-Saldaña, Marcela Rodriguez-Flores, Carlos Cantú-Brito, Jesús Aguirre-Garcia

**Affiliations:** ^1^Resultados Médicos, Desarrollo e Investigación, SC, Boulevard Valle de San Javier, Pachuca Hidalgo 04286, Mexico; ^2^Obesity and Eating Disorders Clinic, Instituto Nacional de Ciencias Médicas y Nutrición “Salvador Zubirán”, SS. Vasco de Quiroga No. 15, Colonia Sección XVI, Tlalpan 14000, Mexico; ^3^Neurology Department, Instituto Nacional de Ciencias Médicas y Nutrición “Salvador Zubirán”, SS. Vasco de Quiroga No. 15, Colonia Sección XVI, Tlalpan, Mexico City 14000, Mexico; ^4^Department of Pathology, Hospital General de México, SS. Dr. Balmis 148, Colonia, Doctores, Mexico City 16726, Mexico

## Abstract

*Objective*. To examine the frequency and patterns of association of cardiovascular risk factors with atherosclerosis in five different arterial territories at post-mortem in Mexico City. *Methods*. We obtained five arterial territories arteries (circle of Willis, coronary, carotid, renal, and aorta) of 185 men and women 0 to 90 years of age who underwent autopsy at the Medical Forensic Service of Mexico City. We determined the prevalence and extent of atherosclerotic lesions by histopathology according to the classification of the American Heart Association as early (types I–III) and advanced (types IV–VI), and according to the degree of stenosis and correlated with cardiovascular risk factors. *Results*. Atherosclerotic lesions were identified in at least one arterial territory in 181 subjects (97.8%), with involvement of two ore more territories in 178 subjects (92.2%). Advanced lesions were observed in 36% and 67% of subjects under 15 and between 16 and 35 years, respectively. Any degree of atherosclerosis was associated with the presence of diabetes mellitus, hypertension, overweight, obesity, and smoking, and to a greater extent with the presence of two or more risk factors (*P* < 0.001). However, emerging and advanced athersoclerosis was observed in 53% and 20% people with no risk factors. *Conclusions*. The study shows a high prevalence of atherosclerosis in all age groups and both sexes. There is considerable development of atherosclerotic disease in subjects without known risk factors.

## 1. Introduction 

Cardiovascular disease is the main cause of disability and premature death worldwide [1] and is projected to remain the leading cause of death. An estimated 17.5 million people died from this cause in 2005, representing 30% of all deaths in the world; of these, 7.6 million were caused by coronary heart disease and 5.7 million by stroke. In low and middle income countries, cardiovascular diseases have become an emerging issue for several reasons: [1] over 80% of the world's deaths for CVD occur in low and middle income countries; [2] people in these countries are more exposed to risk factors leading to CVD and other noncommunicable diseases and are less exposed to prevention efforts than people in high income countries; [3] in these countries, including Mexico, people who suffer from CVD have less access to effective and equitable health care services which respond to their needs [2]. Hence, this group of diseases greatly contributes to the rising costs of health care in the world, and a major public-health challenge, especially for low-income countries [3].

In the 1950s, pathologists began to compare atherosclerosis among populations from different countries [4–6], in order to relate differences in living and environmental conditions to preclinical lesions found at autopsy. As a result, the natural history of atherosclerosis started to be clarified from reports of postmortem studies of young men killed in the Korean and Vietnam wars [7, 8] and from the study of arterial samples of people dying from noncardiovascular causes [9]. These investigations also contributed to document the relationship of risk factors to atherosclerotic lesions, including obesity, hypercholesterolemia, high blood pressure, and smoking [10]. These studies documented that early changes of atherosclerosis were related to the presence of fatty streaks in the intima [11] and the occurrence of these changes at a very early age [12]. In the late 1980s, Stary described the natural history of atherosclerosis in five groups [13], including microscopic changes not previously described, and afterwards, Fuster associated and proposed a classification of coronary atherosclerosis in five stages, based on an updated view of its pathogenesis [14, 15].

The epidemiology and study of the natural history of atherosclerosis in Mexico are recent and scarce: in 1960, Brandt, Pérez-Tamayo, and Ontiveros reported the analysis of 2,000 necropsy studies at a public general hospital in Mexico City, in which they found changes compatible with atherosclerosis in 4.9 percent of the cases [16]; most of these lesions were classified as mild, and only 2.0% were classified as severe. Also in that year, Peterson et al. published a report about the prevalence of carotid thrombosis on a small sample of 39 necropsy studies [17]. In a follow-up study, Escobar reported a 19.1 percent prevalence of atherosclerosis at the circle of Willis among 1,138 necropsy samples [18]. Finally, in a landmark investigation, Cueto García et al. reported the results of the analysis of 167 coronary artery and aortic samples collected from 167 men and 7 women aged between 12 and 64 years, who died from noncardiovascular causes in Mexico City [19–23]. This historical study showed that by comparison with preexisting beliefs, coronary atherosclerosis was very frequent: fatty streaks were found in all of the cases, and the occurrence of fibrolipid plaques was observed at 15 years of age in the thoracic segment and at 19 years of age in the abdominal aorta [22, 23]. Limitatios to this landmark studies are a minority of arterial samples from women, absence of arterial samples from children and the elderly, and limiting the analysis to the coronary and aortic segments.

The main objective of our study was to investigate the frequency of atherosclerotic lesions in a variety of arterial territories of persons undergoing autopsy in Mexico City, in order to ascertain the changes in the frequency and severity of lesions over the last fifty years. Secondary objectives included the comparative analysis of atherosclerotic lesions by age groups and gender and the magnitude of changes according to the reported prevalence of cardiovascular risk factors.

## 2. Methods

Arteries of five different arterial territories were taken from 185 cadavers of men and women aged from 0 to 90 years who died of violent causes and underwent necropsy studies in the Medical Forensic Center in Mexico City from November 2004 to September 2007. The study was approved by the ethical review board of Tribunal Superior de Justicia of Mexico City. Arteries were taken only if authorized by the family. They were not taken for examination in the following circumstances: when tissues have suffered advanced damage that precluded macroscopic or microscopic analyses, when the corpse arrived more than 36 hours after death, in cases with previously reported congenital heart disease.

A survey including sociodemographic and atherosclerosis risk factors was filled out whenever there was family or related people to provide information. In the cases of unknown cadavers, only anthropometric data and causes of death were registered.

The following arterial territories were studied: circle of Willis, right and left carotid arteries, right and left coronary arteries, abdominal aorta, and right and left renal arteries. The circle of Willis was obtained by excision of the basilar artery at the level of the midbrain and then separated manually following the posterior cerebral arteries and then the middle cerebral artery; finally the anterior cerebral arteries were excised 2 cm above the anterior communicating artery. The right and left common carotid arteries were removed. Photographs were taken of representative lesions. The coronary arteries were obtained after fixation of the base of the heart in 10% formalin for 24 hours. The left and right coronary arteries were then removed from their origin at the sinus of Valsalva to its branches; epicardial fat was removed and sections of the following segments were sent for staining: left anterior descending artery, left circumflex artery, diagonal or septal branch of the left coronary artery, the posterior descending artery, the sinus node artery, and the marginal branch of the right coronary artery. The aorta was removed from below the inferior mesenteric artery to 2 cm above the iliac bifurcation including 2 cm of the renal arteries.

After removal, tissues were fixed in 10% formalin for 24 hours, adipose adventitious tissue was removed from the carotid, aortic, and renal arteries by sharp dissection and they were opened longitudinally for identification. They were prepared for microscopic examination by paraffin embedding and staining of sections with the most advanced macroscopic lesions. Three different sections from the circle of Willis were dissected, including the areas where lesions were evident and submitted for staining. The sections for microscopy were 1 cm in length cut transversely of each arterial territory.

Photographs were taken from representative microscopic and macroscopic lesions.

The statistical analysis was made with the data of 185 cases with microscopic evaluation. The prevalence of atherosclerosis was established by the number of cases with existing atherosclerotic lesions. The extent of atherosclerosis was established by the number of territories affected in each case. The degree of atherosclerosis was established according to defining criteria from the Council of Atherosclerosis of the American Heart Association [24, 25] and according to the degree of the occlusion whenever measurable. Data are presented as means (SD) for continuous variables and percentages for qualitative variables. Differences in prevalence, extent, and degress of aterosclerosis were analyzed according to causes of death and with cardiovascular risk factores with the Student's *t*-test and chi square test.

## 3. Results

**Figure 1:**
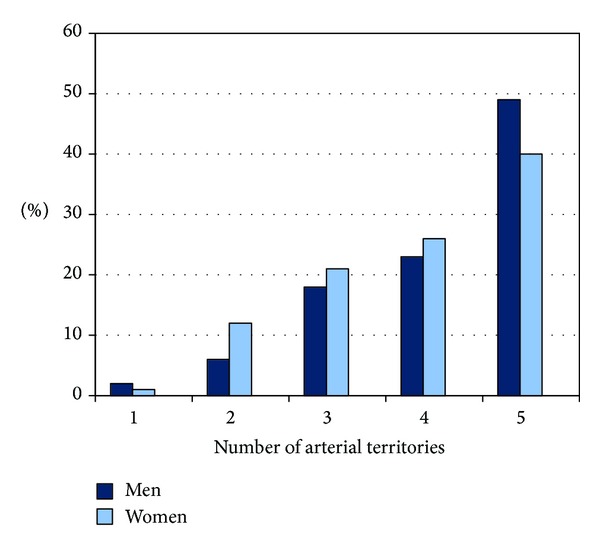
Number of arterial territories (intracranial, carotid, coronary, renal, and aorta) with atherosclerosis according to gender.

**Figure 2:**
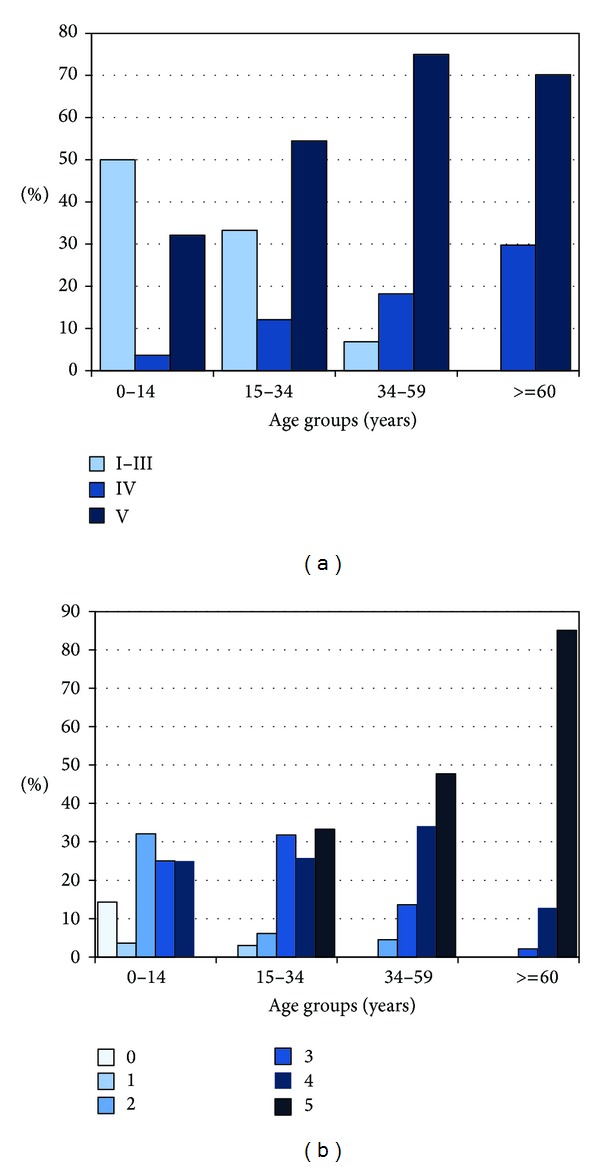
(a) Atherosclerosis severity (AHA pathological classification) in at least one of the five studied arterial territories (intracranial, carotid, coronary, renal, and aorta) according to age. (b) Number of atherosclerotic arterial territories (intracranial, carotid, coronary, renal, and aorta) according to age.

The study sample included 78 women, median age of 30 years (IQR 19–63 years); and 107 men, median age of 35 years (IQR 23–60 years), who were classified in four age groups: group 1: 1–14 years (*N* = 28), group 2: 15–34 years (*N* = 66), group 3: 35–59 years (*N* = 44), and group 4: 60 years and older (*N* = 46).  Table 1 describes the demographic data, causes of death, and arterial territories available for pathologic analysis. Atherosclerosis was identified in at least one of the five arterial territories in 184 individuals (97.8%) and involvement of two or more territories was observed in 178 (92.2%). The four subjects without atherosclerosis were younger than one year old.  Figure 1 shows the number of arterial territories with atherosclerosis according to gender; although the frequency of atherosclerosis was similar in both men (97.2%) and women (98.7%), involvement of the five territories tended to be more common in men (48.6% versus 39.7%; *P* = 0.23). As expected, severity of atherosclerosis and number of involved arterial territories increased with age as shown in Figures 2(a) and 2(b); however, the rate of atherosclerosis grades IV and V was not uncommon in young subjects: raise plaques were observed in 36% and 67% of subjects younger than 15 and 35 years old, respectively. Also, involvement of three to five territories occurred in around 25% and 30% of young subjects of these age groups, respectively.

Table 2 describes the prevalence of atherosclerotic lesions by arterial territory and severity (AHA pathological classification) according to age groups. Coronary arteries, aorta, and carotid arteries were the most common arterial territories involved by atherosclerosis (98, 97%, and 86%, resp.), whereas the renal (71%) and intracranial (60%) arteries were least frequently involved. Severity of atherosclerosis corresponds usually to grades from III to V, mainly in coronary, intracranial, and renal arteries. Indeed, among raise plaques (types IV-V), some grades of arterial stenosis ≥5% of lumen reduction were documented in 50% of subjects in coronary arteries, 17% in renal arteries, 15% in arteries of Willis polygon, 11% in carotid arteries, and 3% of subjects at the level of aorta. The remakably high frequency of arterial stenosis in coronary arteries is evident since young age (38% among people 15–34 years of age).  Table 3 shows the demographic data and aterosclerosis severity according to the cause of death.

Information regarding vascular risk factors was available in 122 subjects.  Table 4 describes the prevalence of demographic data, causes of death, and vascular risk factors in relation to severity of atherosclerosis, excluding the four infants without evidence of atherosclerotic lesions. Median age of subjects with severe atherosclerosis was twofold compared with mild atherosclerosis (*P* < 0.001), but there were no differences between gender. Atherosclerosis was associated with diabetes, hypertension, overweight (BMI 25–29.9), obesity (by BMI ≥ 30), and abdominal obesity when criteria of IDF is used (men ≥ 90 cm; female ≥ 80 cm) and mainly with the presence of two or more risk factors (*P* < 0.001). However, mild and severe atheroscleroses were observed in 53% and 20% of persons without traditional risk factors.

## 4. Discussion

The incidence of chronic-degenerative diseases has increased dramatically in Mexico [26]. Cardiovascular disorders have been leading factors, as reflected by sustained increments in morbidity, mortality, and prevalence [27, 28]. Atherosclerosis is the most important feature of macrovascular disease, and its epidemiologic analysis is a topical issue [29]. The importance of aterosclerosis as a worlwide health problema, and its increasing frequency in developed countries, were not recognized until the 20th century. Concurrently, great advances have occurred to clarify its pathophysiology, from a variety of individual mechanisms [30–42] to an integrating model, with inflammation as the key component [43].

The role of autopsy in present day medical practice, teaching, and research has been debated [44]. Population based epidemiological studies of atherosclerosis using autopsy material have become increasingly less frequent, due to several factors, including a decline in autopsy rates [45], and the use of noninvasive methods to assess atherosclerotic lesions in the population [46]. Epidemiological studies based on autopsy material, however, are still used to make longitudinal comparisons of the level of atherosclerosis in populations from countries with a long term established cardiovascular burden [47]. In recent years, the study of atherosclerosis in autopsy studies is still a resource to investigate the incidence of cardiovascular disease in countries where information about atherosclerosis was either unavailable or was considered uncommon, due to environmental influences or protective behavioral and socioeconomic factors that delay atherogenesis [48]. For instance, Bertomeu et al. consecutively collected specimens from 65 young and healthy people aged between 12 and 35 years who died from external causes in Barcelona. Their findings showed the presence of fibrous plaques in 34% of coronary specimens in men, and in 22% in women. Advanced lesions were more common in older men, and in people with higher serum cholesterol, while no differences were found with respect to adiposity or smoking with the frequency of plaques [49].

Our study is relevant to the following several reasons. (1) To illustrate the increasing contribution of noncommunicable diseases to morbidity and mortality in middle income countries [50]. As already mentioned, 80 percent of the burden of cardiovascular disease currently occurs in countries like Mexico [2, 3], where ischemic heart disease and stroke were the second and third leading causes of overall mortality in 2008, with 59,579 and 30,212 deaths, respectively, which collectively represent 16.7% of all the deaths in the country. Diabetes, another major causative factor of atherosclerosis, has been the first cause of general mortality in Mexico, with 75,572 deaths occurring in 2008, which correspond to 14.0% of the general mortality. Combined, the three main causes of mortality in Mexico represent one-third of all the deaths in the country [51]. (2) In the absence of resources to carry out large, representative population-based analysis on the epidemiology of atherosclerosis in middle and low income countries by noninvasive methods, autopsy studies in people dying from noncardiovascular causes will continue to be a useful strategy to assess the status of these health problems. (3) To compare the results of our study with a previous analysis, from the autopsy studies published by Cueto García et al. in the 1980s [19–23]. The comparison shows clear differences over time: albeit the findings from the original studies already documented the presence and severity of atherosclerosis with advancing age, the prevalence of coronary atherosclerosis is lower in the first studies, ranging from 1.6% in the 15–19 to 76% in the 45–49 age group [20]. By comparison, the results of our study documented a high prevalence of coronary atherosclerotic lesions in every age group, albeit with different levels of severity, ranging from 75.5% in the 1–14 year group to 100.0% in each of the three remaining age groups. Significant differences in the prevalence of advanced and stenotic lesions were noted in our study, ranging from 11.5% to 3.3% in the 15–34 year olds, to 77.8% and 46.7% in samples from people of 60 years and older. [4] Except for the study of lesions in the descending aorta, the previous study of atherosclerosis in Mexico did not include the analysis of additional arterial territories. We have not found a previous study in the history of the natural history of atherosclerosis in which samples from several arterial territories were included. The information that we collected could be submitted to further analysis, to determine the frequency of associations of atherosclerotic lesions in different arterial regions, in support of the concept that cardiovascular disease is a localized manifestation of atherosclerosis. Additionally, we are currently analyzing the likelihood of associations between preexisting cardiovascular risk factors, with the location, number of territories, and burden of atherosclerosis.

Limitations of our study include the difficulty of collecting all the samples within a restricted time frame, because, compared to adult males, the number of samples from children, women, and the elderly reaching forensics departments is smaller. A selection bias results from the social problems involved in the demise of the population under study, the absence of samples from indigenous Mexican populations, and the lack of genetic analysis in these samples. Some of these shortcomings are currently being reassessed in the study sample, and we expect to include them in a follow-up study.

In conclusion, the analysis of arterial samples of people dying from noncardiovascular causes in Mexico City showed a high prevalence of atherosclerosis in each of the five arterial territories under study. Mild lesions were first noticed in the carotid and aorta, while advanced, stenotic lesions were more common in the coronaries. Similarities in the prevalence and progression of lesions were observed in the intracranial and renal vessels, and comparisons of the current study of coronary lesions with a previous analysis document the advance of atherosclerosis in men and women in Mexico, starting in infancy.

## Figures and Tables

**Table 1:** Demographic data, death causes, and distribution of arterial territories available for pathological analysis of the 185 subjects.

Gender (%)	
Male	107 (57.8)
Female	78 (42.2)
Age, years; median (IQR)	34 (20–70)
Causes of death (%)	
Accident	73 (39.5)
Homicide	42 (22.7)
Asphyxia	13 (7.0)
Nonvascular illness	43 (23.2)
Pneumonia	18 (9.7)
Other	25 (13.5)
Vascular	14 (7.6)
Acute myocardial infarction	7 (3.8)
Intracerebral hemorrhage	3 (1.6)
Pulmonary thromboembolism	2 (1.1)
Acute aortic rupture	2 (1.1)
Arterial territories available for pathological analysis of 185 subjects (%)	
Coronary	
Right coronary artery	177 (95.7)
Left coronary artery	179 (96.7)
Carotid	
Right	185 (100)
Left	181 (97.8)
Intracranial (Willis polygon)	179 (96.7)
Renal	
Right	169 (91.4)
Left	170 (91.9)
Aorta	177 (95.7)

**Table 2:** Prevalence of atherosclerotic lesions by arterial territory and severity (AHA pathological classification) according to age groups.

Arterial territory and atherosclerosis grade	1–14 y	Age groups 15–34 y	35–59 y	≥60 y
Intracranial (%)	*N* = 24	*N* = 65	*N* = 43	*N* = 47
None	17 (70.8)	34 (52.3)	16 (37.2)	5 (10.6)
Grades I-II	1 (4.2)	0	0	0
Grade III	5 (20.8)	26 (40.0)	20 (46.5)	15 (31.9)
Grades IV-V	1 (4.2)	5 (7.7)	7 (16.3)	27 (57.4)
Stenosis*	0	2 (3.0)	4 (9.3)	22 (46.8)

Carotid (%)	*N* = 28	*N* = 66	*N* = 44	*N* = 47
None	16 (57.1)	8 (12.1)	1 (2.3)	0
Grades I-II	6 (21.4)	14 (21.2)	1 (2.3)	0
Grade III	6 (21.4)	38 (57.6)	24 (54.5)	14 (29.8)
Grades IV-V	0	6 (9.1)	18 (40.9)	33 (70.2)
Stenosis*	0	0	7 (15.9)	14 (29.8)

Coronary (%)	*N* = 28	*N* = 63	*N* = 43	*N* = 46
None	6 (21.4)	0	0	0
Grades I-II	2 (7.1)	1 (1.6)	0	0
Grade III	12 (42.9)	21 (33.3)	4 (9.3)	2 (4.3)
Grades IV-V	8 (28.6)	41 (65.1)	39 (90.7)	44 (95.7)
Stenosis*	1 (3.6)	24 (38.1)	23 (53.5)	41 (89.1)

Renal (%)	*N* = 27	*N* = 61	*N* = 40	*N* = 45
None	22 (81.5)	24 (39.3)	5 (12.5)	0
Grades I-II	0	1 (1.6)	1 (2.5)	0
Grade III	5 (18.5)	29 (47.5)	22 (55)	10 (22.2)
Grades IV-V	0	7 (11.5)	12 (30.0)	35 (77.8)
Stenosis*	0	2 (3.3)	6 (15.0)	21 (46.7)

Aorta (%)	*N* = 28	*N* = 63	*N* = 41	*N* = 46
None	6 (21.4)	0	0	0
Types I-II	3 (10.7)	14 (22.2)	4 (10.0)	0
Grade III	1 (3.6)	5 (7.9)	25 (62.5)	43 (93.5)
Types IV-V	0	6 (9%)	18 (41%)	33 (70%)
Stenosis*	0	0	0	5 (10.9)

*All arterial stenosis are >25% of lumen reduction and correspond to subjects with plaques types IV-V.

**Table 3:** Demographic data and atherosclerosis severity according to causes of death.

	Asphyxia *N* = 13	Accident and homicide *N* = 115	Nonvascular illness *N* = 43	Vascular disease *N* = 14	*P*
Age, median (IQR)	14 (1–28.5)	31 (20–50)	41 (23–66)	68 (56–70)	<0.001
Gender (%)					
Female	9 (69.2)	45 (39.1)	22 (51.2)	2 (14.3)	0.01
Male	4 (30.8)	70 (60.9)	21 (48.8)	12 (85.7)	
Atherosclerosis severity by AHA grade					
None	1 (7.7)	0	3 (7.0)	0	<0.001
I–III	8 (61.5)	28 (24.3)	3 (7.0)	0	
IV-V	4 (30.8)	87 (75.7)	37 (86.0)	14 (100.0)	
Arterial territories with atherosclerosis					
0-1	3 (23.1)	1 (0.9)	3 (7.0)	0	<0.001
2-3	7 (53.8)	34 (29.6)	6 (14.0)	3 (21.4)	
4-5	3 (23.1)	80 (69.6)	34 (79.1)	11 (78.6)	
Atherosclerosis with stenosis >25%					
No	11 (84.6)	62 (53.9)	17 (39.5)	0	<0.001
Yes	2 (15.4)	53 (56.1)	26 (60.5)	14 (100.0)	

**Table 4:** Demographic data and vascular risk factors according to atherosclerosis severity.

	Atherosclerosis severity by grade		*P*	No. of arterial territories with atherosclerosis		*P*	Atherosclerosis with stenosis ≥25%		*P*
	I–III *N* = 39	IV-V *N* = 142		1–3 *N* = 53	4-5 *N* = 128		No *N* = 86	Yes *N* = 95	
Gender (%)									
Male	21 (53.8)	83 (58.5)	0.60	27 (50.9)	77 (60.2)	0.25	47 (54.7)	57 (60.0)	0.46
Female	18 (46.2)	59 (41.5)		26 (49.1)	51 (39.8)		39 (45.3)	38 (40.0)	
Age, median (IQR)	19 (6–24)	41 (28–65)	<0.001	20 (11–28)	45 (29–66)	<0.001	23 (14–33)	58 (34–70)	<0.001
Vascular risk factors (%)	*n* = 32	*n* = 86		*n* = 41	*n* = 77		*n* = 65	*n* = 57	
Hypertension	0	17 (19.8)	0.006	2 (4.9)	15 (19.5)	0.03	2 (3.1)	15 (26.3)	<0.001
Diabetes mellitus	0	12 (14.0)	0.03	1 (2.4)	11 (14.3)	0.05	1 (1.6)	11 (19.3)	0.002
Smoking	6 (18.8)	24 (27.9)	0.31	9 (22.0)	21 (27.3)	0.52	16 (26.2)	14 (24.6)	0.83
Alcohol use	8 (25.0)	24 (27.9)	0.75	8 (19.5)	24 (31.2)	0.17	18 (29.5)	14 (24.6)	0.54
BMI	*n* = 21	*n* = 84		*n* = 29	*n* = 76		*n* = 49	*n* = 58	
Overweight	6 (28.6)	31 (36.9)	0.13	6 (20.7)	31 (40.8)	0.006	14 (28.6)	23 (39.7)	0.03
Obesity	3 (14.3)	18 (21.4)	0.03	1 (6.9)	19 (25.0)	0.005	5 (10.2)	16 (27.6)	0.004
Abdominal obesity	*n* = 33	*n* = 130		*n* = 45	*n* = 118		*n* = 74	*n* = 89	
Men ≥ 102 cm; female ≥ 88 cm	4 (12.1)	38 (29.2)	0.04	8 (17.8)	34 (28.8)	0.15	12 (16.2)	30 (33.7)	0.01
Men ≥ 90 cm; female ≥ 80 cm	9 (27.3)	66 (50.8)	0.01	17 (37.8)	58 (49.2)	0.19	23 (31.1)	52 (58.4)	<0.001
Number of risk factors	*n* = 32	*n* = 86		*n* = 41	*n* = 77		*n* = 65	*n* = 57	
None	17 (53.1)	17 (19.8)	0.001	21 (51.2)	13 (16.9)	<0.001	30 (46.2)	8 (14.0)	<0.001
1-2	13 (40.6)	44 (51.2)		15 (36.6)	42 (54.5)		30 (46.2)	27 (47.4)	
3–5	2 (6.3)	25 (29.1)		5 (12.2)	22 (28.6)		5 (7.7)	22 (38.6)	
